# Supplementary material for: On-chip mid-infrared photothermoelectric detectors for full-Stokes detection
Source: Nat Commun. 2022 Aug 5;13:4560. doi: 10.1038/s41467-022-32309-w (PMC9356042; doi:10.1038/s41467-022-32309-w)
Supplement: Supplementary file 1 — Supplementary Information [file 41467_2022_32309_MOESM1_ESM.pdf]

Supplementary Information

for

On-chip mid-infrared photothermoelectric detectors for full-Stokes detection

Mingjin Dai[1], Chongwu Wang[1], Bo Qiang[1], Fakun Wang[1], Ming Ye[1], Song Han[1], Yu Luo[1], Qi Jie Wang[1,2]

[1]School of Electrical and Electronic Engineering, Nanyang Technological University, Singapore 639798, Singapore.

[2]Centre for Disruptive Photonic Technologies, Division of Physics and Applied Physics, School of Physical and Mathematical Sciences, Nanyang Technological University, Singapore 637371, Singapore.

Corresponding Author: qjwang@ntu.edu.sg; luoyu@ntu.edu.sg

Contents:

Supplementary Notes 1-2

Supplementary Figures 1-21

Supplementary Tables 1-3

Supplementary References 1-38

Supplementary Note 1: State of polarization dependent absorption of the chiral metamaterials

As shown in the main manuscript, the state of polarization dependent absorption can fit well with a cosine function of the azimuthal angle θ with a weighted shift factor given by ellipticity angle φ. In detail, the extraction process of the coefficients (a_1, a_2, b_1, b_2) in the equation is as follows:

Firstly, by fitting the azimuthal angle θ dependent absorption with a fixed ellipticity angle φ using a cosine function, a series of coefficients pairs (*a*, *b*) can be obtained. Secondly, the coefficient *a* as a sine function of ellipticity angle φ is fitted and two coefficients (a_1 and a_2) representing the constant component and the amplitude of the φ-resolved component can be obtained. Thirdly, the coefficient *b* as a cosine function of ellipticity angle φ is fitted and two coefficients (b_1 and b_2) representing the constant component and the amplitude of the φ-resolved component can also be obtained.

Supplementary Note 2: Derivation of the expressions for calculation of geometrical ellipse parameters

As we designed, the photovoltage output of each port is polarization dependent. Particularly, the photovoltage outputs of Port 1 and Port 2 are linear and circular polarization dependent, and the photovoltage output of Port 3 is only circular polarization dependent. The general expression of the photovoltages for each part in our designed device can be expressed as:

$$V_{ph} = L_i \sin(2\theta) + C_i \tan(\varphi) \quad (1)$$

where, $L_i \cdot \sin(2\theta)$ is the linear-polarization-resolved (θ-resolved) photovoltage component, and $C_i \cdot \tan(\varphi)$ is the circular-polarization-resolved (φ-resolved) photovoltage component. No constant background photoresponse is available due to the bipolar responses of our devices. In detail, the expression of each port in our three-ports device can be expressed as:

$$P_1 = (L_1 \sin(2(\theta + 45)) + 0.5C_1 \tan(\varphi)) - (L_0 \sin(2\theta) - 0.5C_0 \tan(\varphi)) \quad (2)$$

$$P_2 = (L_2 \sin(2(\theta + 135)) + 0.5C_2 \tan(\varphi)) - (L_0 \sin(2\theta) - 0.5C_0 \tan(\varphi)) \quad (3)$$

$$P_3 = (L_3 \sin(2(\theta)) + C_3 \tan(\varphi)) - (L_0 \sin(2\theta) - C_0 \tan(\varphi)) \quad (4)$$

Here, the coefficients (L_i and C_i) for each port output can be obtained by calibration with the experimental results. Based on the experimental results as shown in Supplementary Figure 19, we can simplify the Eq. 2-4 as:

$$P_1 = (L_1' \sin(2(\theta - 112.5)) + C_1' \tan(\varphi)) \quad (5)$$

$$P_2 = (L_2' \sin(2(\theta - 67.5)) + C_2' \tan(\varphi)) \quad (6)$$

$$P_3 = -C_3' \tan(\varphi) \quad (7)$$

According to fitting results with experimental results, the coefficients can meet relationships as: $L''=L_1'=L_2'$, and $C''=C_1'=C_2'=C_3'$. Therefore, the Eq. 5-7 can be further simplified as:

$$P_1 = (L'' \sin(2\theta - 45) + C'' \tan(\varphi)) \quad (8)$$

$$P_2 = (L'' \cos(2\theta - 45) + C'' \tan(\varphi)) \tag{9}$$

$$P_3 = -C'' \tan(\varphi) \tag{10}$$

Therefore, the azimuthal angle θ and ellipticity angle φ can be calculated as:

$$\theta = \frac{1}{2}\left(\tan^{-1}\frac{P_1+P_3}{P_2+P_3} + 45\right) \qquad \text{when } P_2 < 0 \tag{11}$$

$$\theta = \frac{1}{2}\left(\tan^{-1}\frac{P_1+P_3}{P_2+P_3} + 45\right) + 90 \qquad \text{when } P_2 > 0 \tag{12}$$

$$\varphi = \tan^{-1}\frac{P_3}{C} \tag{13}$$

where only one coefficient (C) is needed to be extracted, which is related to the incident light power.

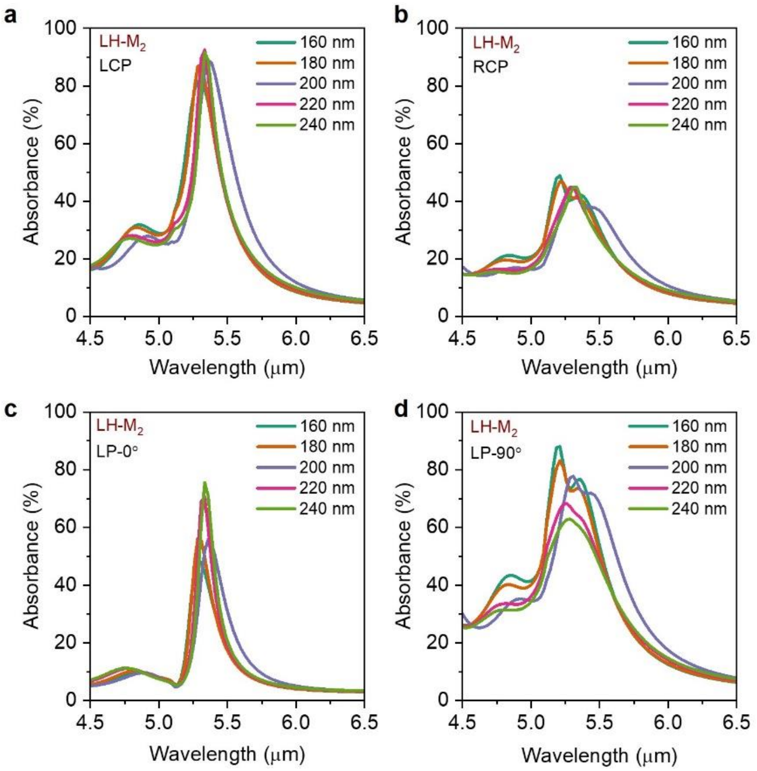

Supplementary Figure 1. Effect of dielectric spacer (Al_2O_3) thickness on the optical absorption of the metamaterials. a-d, The absorption spectrum of LH-M_2 metamaterials with different dielectric spacer thicknesses.

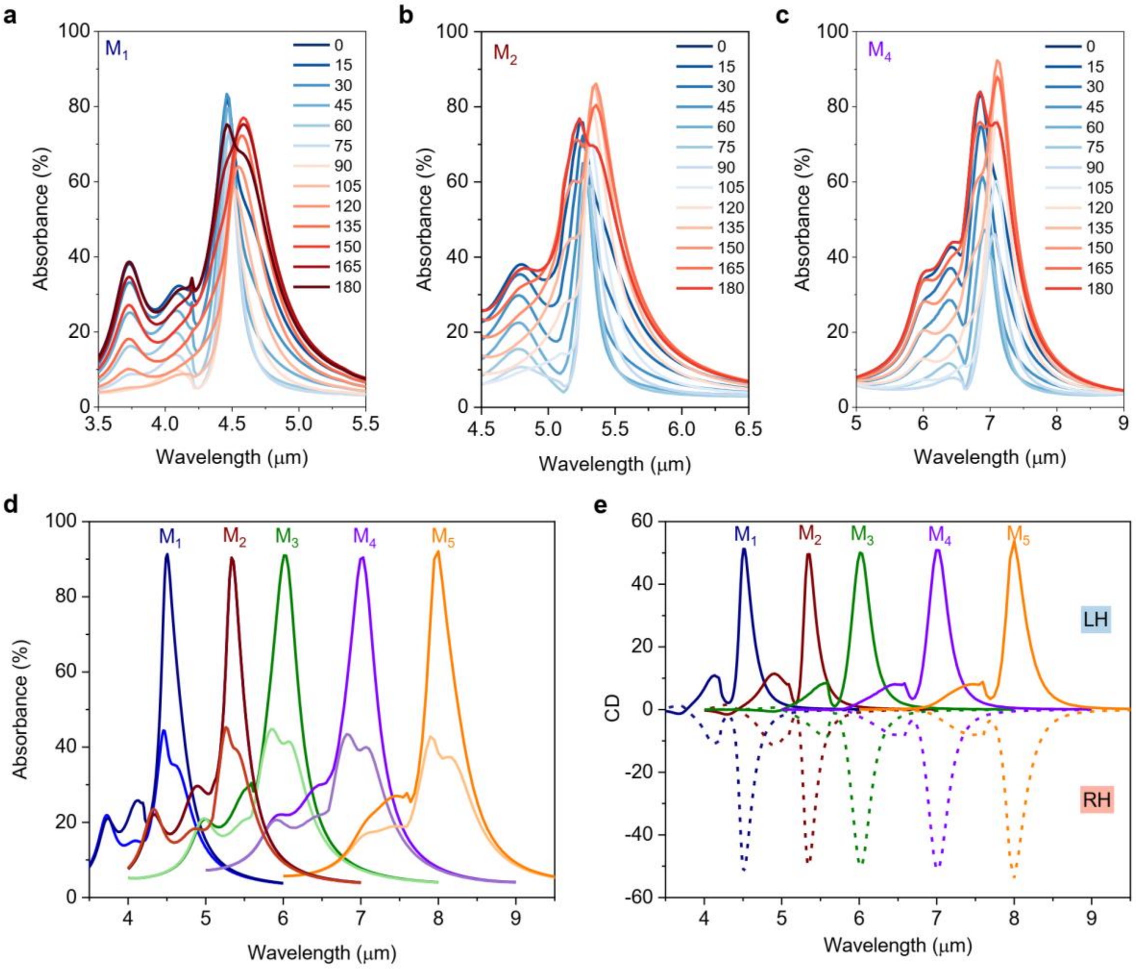

Supplementary Figure 2. Simulated polarization dependent optical absorption of metamaterials. a-c, Linear polarization dependent optical absorption of metamaterial M_1 (**a**) M_2 (**b**), and M4 (**c**). **d**, Simulated optical absorption under LCP and RCP illumination for LH metamaterial with different dimensions (M_1 to M_5). **e**, Corresponding CD as a function of wavelength for both LH and RH metamaterials.

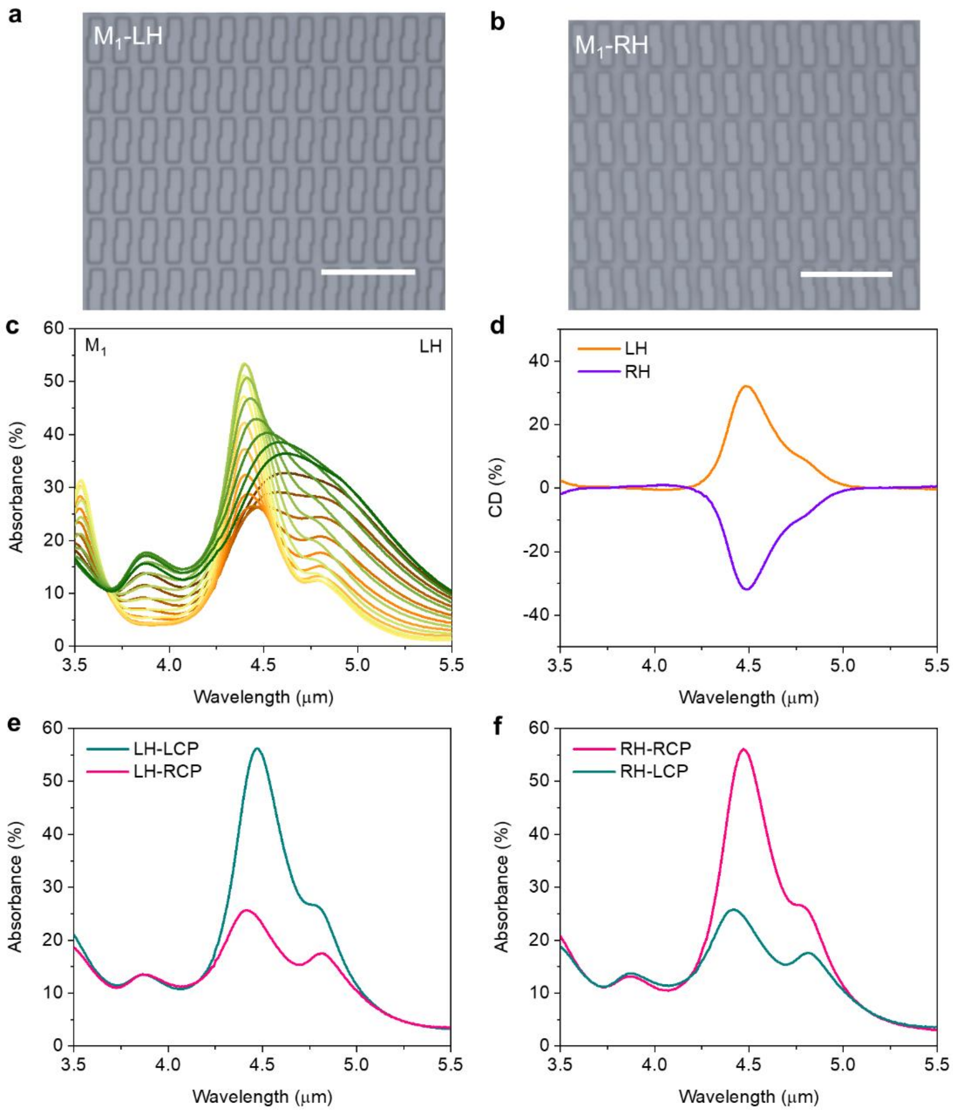

Supplementary Figure 3. Experimental measurement of polarization-dependent optical absorption of metamaterial M_1. a,**b**, Optical images of LH (**a**) and (**b**) RH metamaterials M_1. Scale bar: 10 µm. **c**, Measured linear polarization dependent optical absorption for LH metamaterial. **d**, CD as a function of wavelength for both LH and RH metamaterials M_1. **e**,**f**, Measured optical absorptions under LCP and RCP illumination for LH (**e**) and RH (**f**) metamaterials M_1.

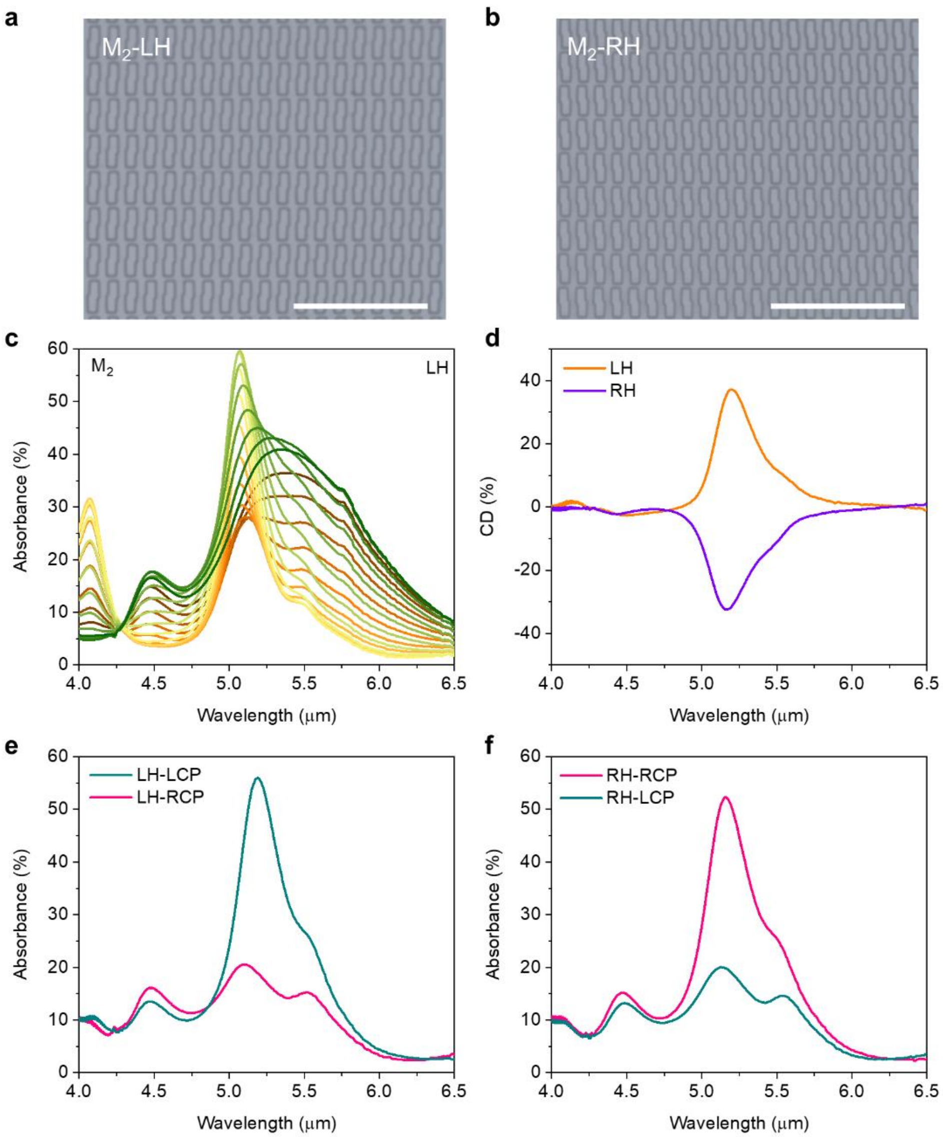

Supplementary Figure 4. Experimental measurement of polarization-dependent optical absorption of metamaterial M_2. a,**b**, Optical images of LH (**a**) and (**b**) RH metamaterials M_2. Scale bar: 20 µm. **c**, Measured linear polarization dependent optical absorption for LH metamaterial. **d**, CD as a function of wavelength for both LH and RH metamaterials M_2. **e**,**f**, Measured optical absorptions under LCP and RCP illumination for LH (**e**) and RH (**f**) metamaterials M_2.

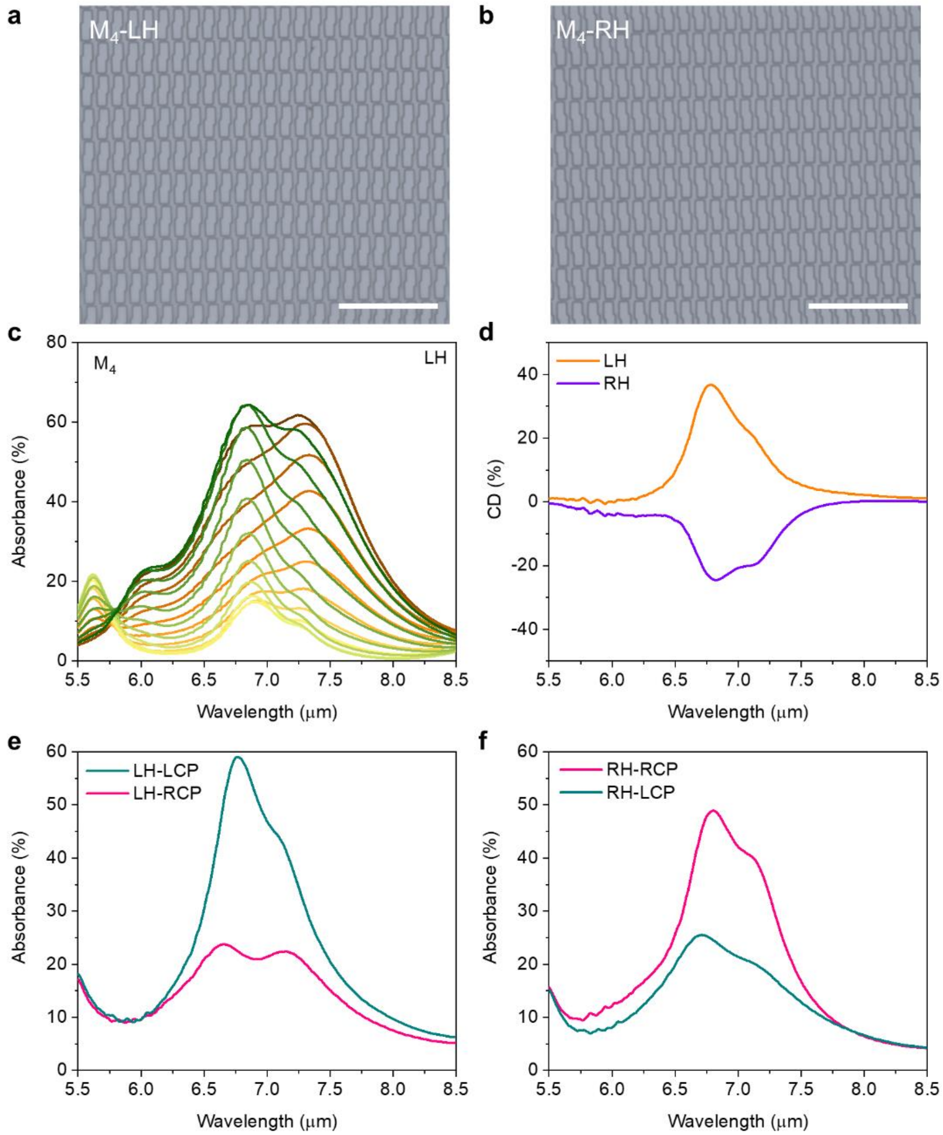

Supplementary Figure 5. Experimental measurement of polarization-dependent optical absorption of metamaterials M_4. a,**b**, Optical images of LH (**a**) and (**b**) RH metamaterials M_4. Scale bar: 20 µm. **c**, Measured linear polarization dependent optical absorption for LH metamaterial. **d**, CD as a function of wavelength for both LH and RH metamaterials M_4. **e**,**f**, Measured optical absorptions under LCP and RCP illumination for LH (**e**) and RH (**f**) metamaterials M_4.

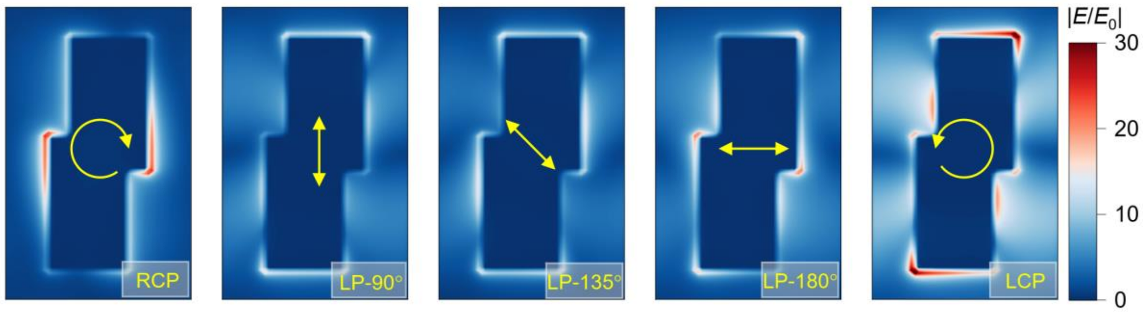

Supplementary Figure 6. Electric field distributions normalized to incident electric field at different polarization status for the LH metamaterial.

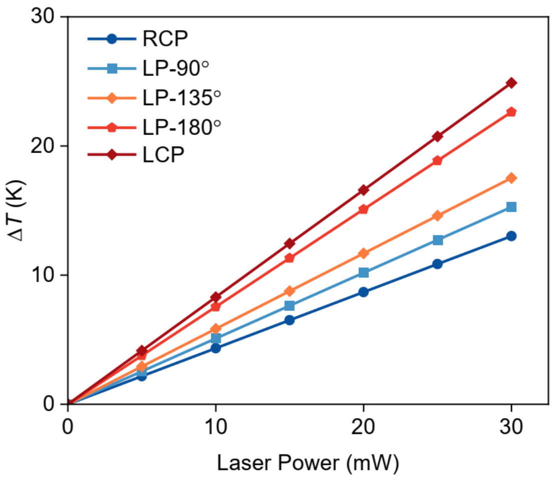

Supplementary Figure 7. Analytical temperature increase Δ*T* versus incident laser power under different polarization status for the LH metamaterial.

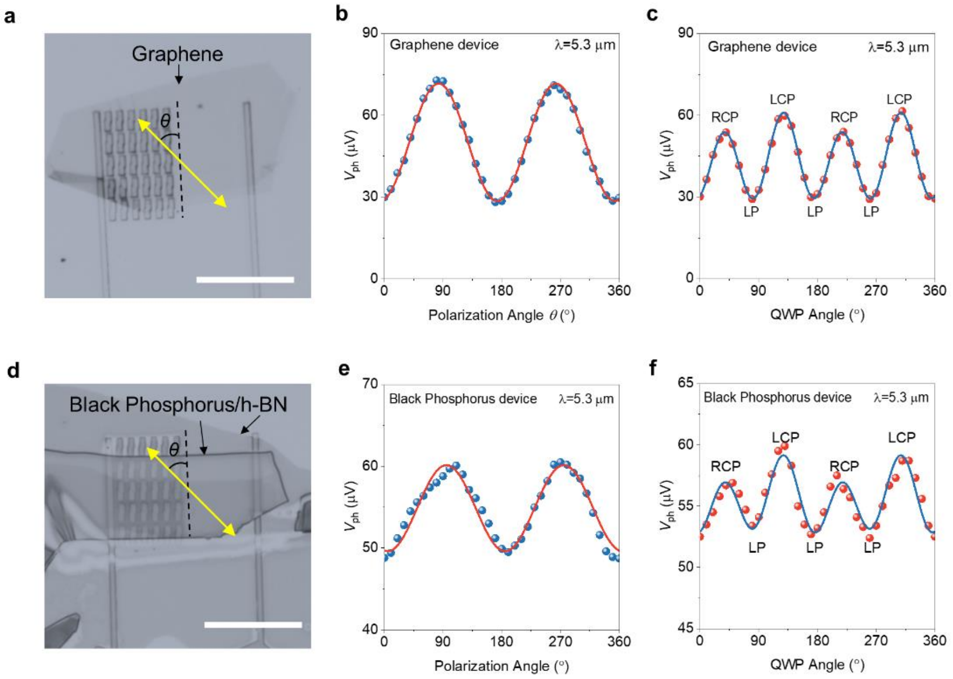

Supplementary Figure 8. HWP and QWP angle dependent photoresponse of Gr and BP device with half-side LH metamaterial pattern for 5.3 µm infrared light, respectively. **a**,**d**, Optical images of Graphene (**a**) and BP (**d**) devices. Scale bars: 20 µm. **b**,**c**, Polarization angle (**b**) and QWP angle (**c**) dependent photoresponse of Graphene device. **e**,**f**, Polarization angle (**e**) and QWP angle (**f**) dependent photoresponse of BP device.

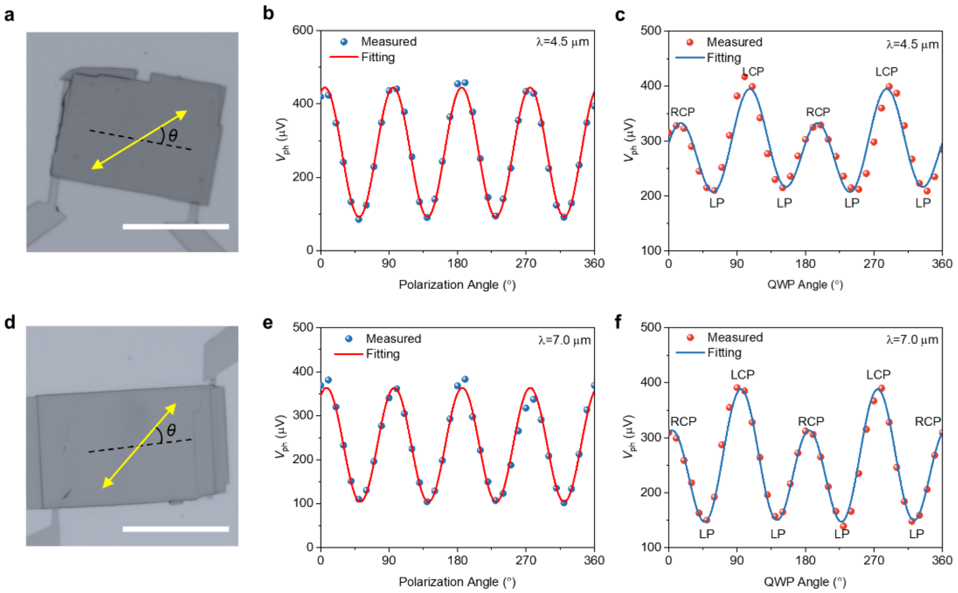

Supplementary Figure 9. HWP and QWP angle dependent photoresponse of $PdSe_2$ device with half-side LH metamaterial pattern for 4.5 and 7.0 μm infrared light, respectively. a,**d**, Optical images of $PdSe_2$ devices for 4.5 (**a**) and 7.0 (**d**) μm detection. Scale bars: 20 μm. **b**,**c**, HWP angle (**b**) and QWP angle (**c**) dependent photoresponse under 4.5 μm infrared light illumination. **e**,**f**, HWP angle (**e**) and QWP angle (**f**) dependent photoresponse under 7.0 μm infrared light illumination.

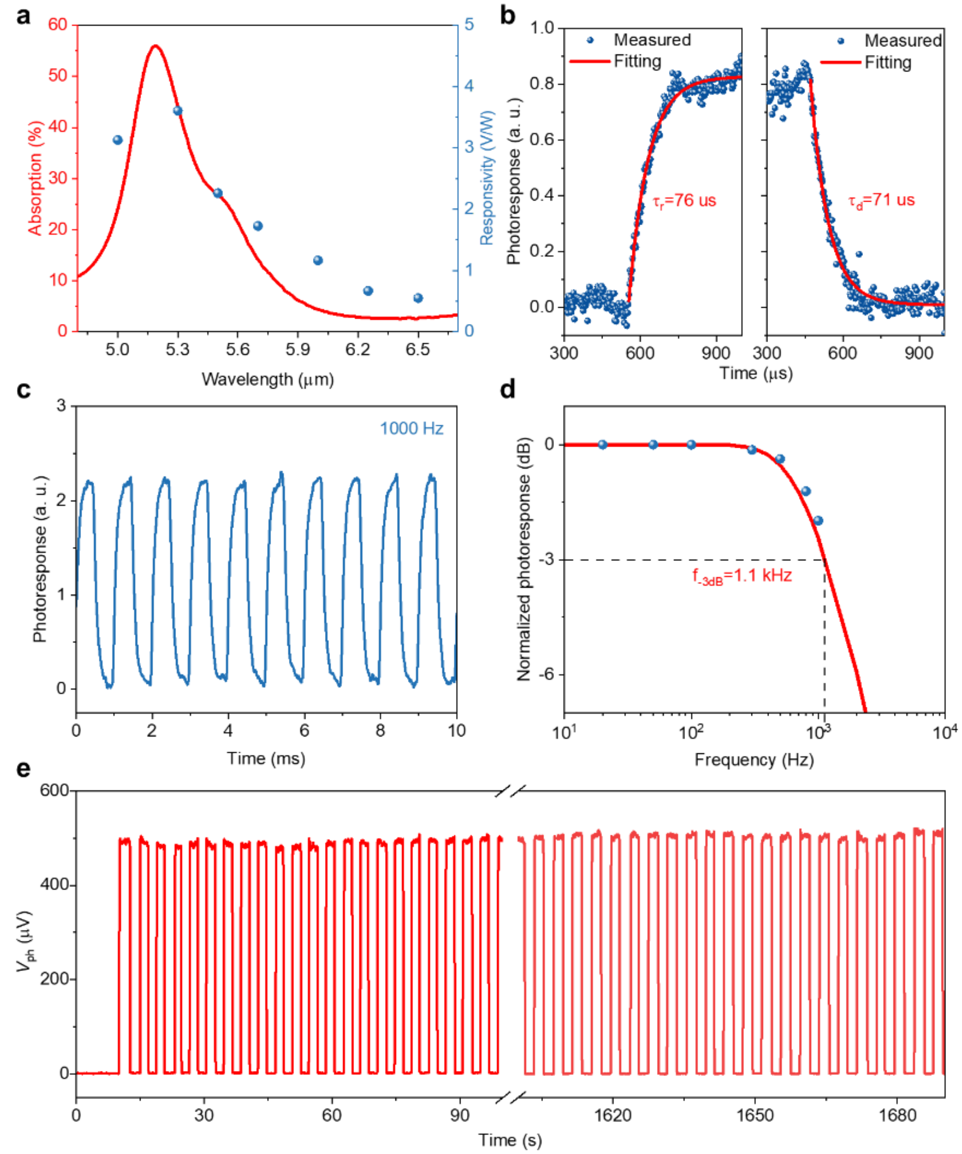

Supplementary Figure 10. Photoresponse speed and stability of $PdSe_2$ device. a, The absorption spectrum (red line) of LH-M_2 metamaterials under linear polarized light illumination and corresponding responsivity (blue dots) of LH-M_2 metamaterials mediated device. The incident laser power is fixed at 20 mW. **b**, Time-resolved photoresponse of a typical $PdSe_2$ device under 5.3 μm laser illumination. The solid lines are the fitting curves by using exponential function, showing a rise and decay response time constant of 76 and 71 μs, respectively. **c**, Measured time-resolved photoresponse response of our device with the illumination signal chopped at 1000 Hz. **d**, Normalized photoresponse as a function of frequency showing a -3dB bandwidth of around 1.1 kHz. **e**, Measured long-term photoresponse showing a good repeatability and stability.

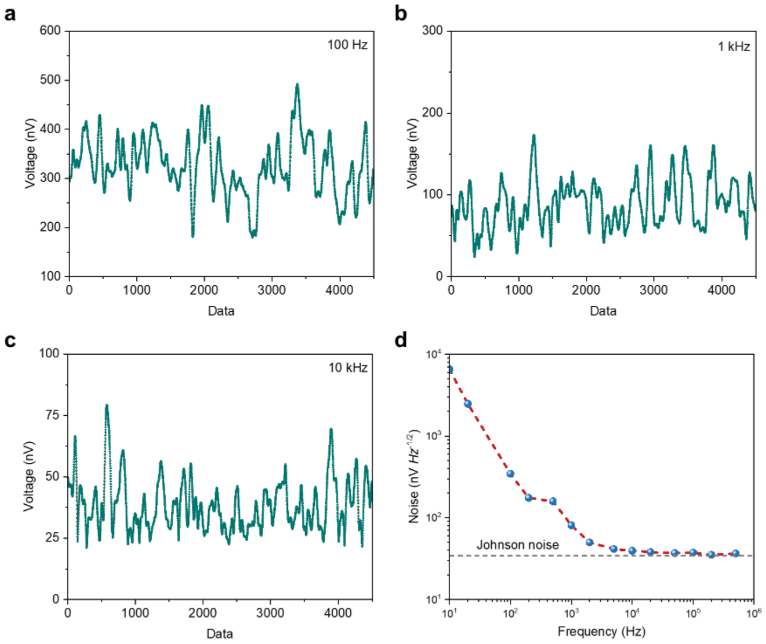

Supplementary Figure 11. Noise measurements. a-**c**, The voltage data measured with a Lock-in amplifier under different internal referenced frequency: 100 Hz (**a**), 1 kHz (**b**), and 10 kHz (**c**). **d**, The spectral density of voltage noise showing a dramatically decrease with frequency and then keeping a constant when the frequency is over around 1 kHz.

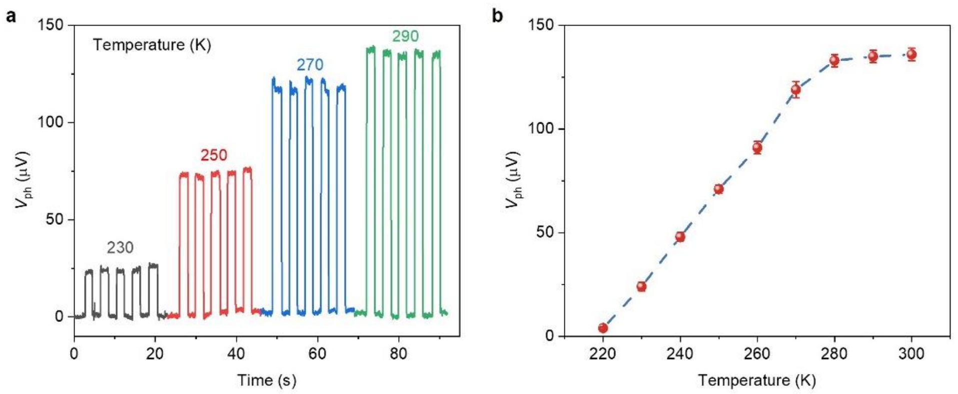

Supplementary Figure 12. Temperature dependent photoresponse. a, The photoresponses at different temperatures. **b**, The temperature-dependent photovoltages with an incident light power of 38 μW.

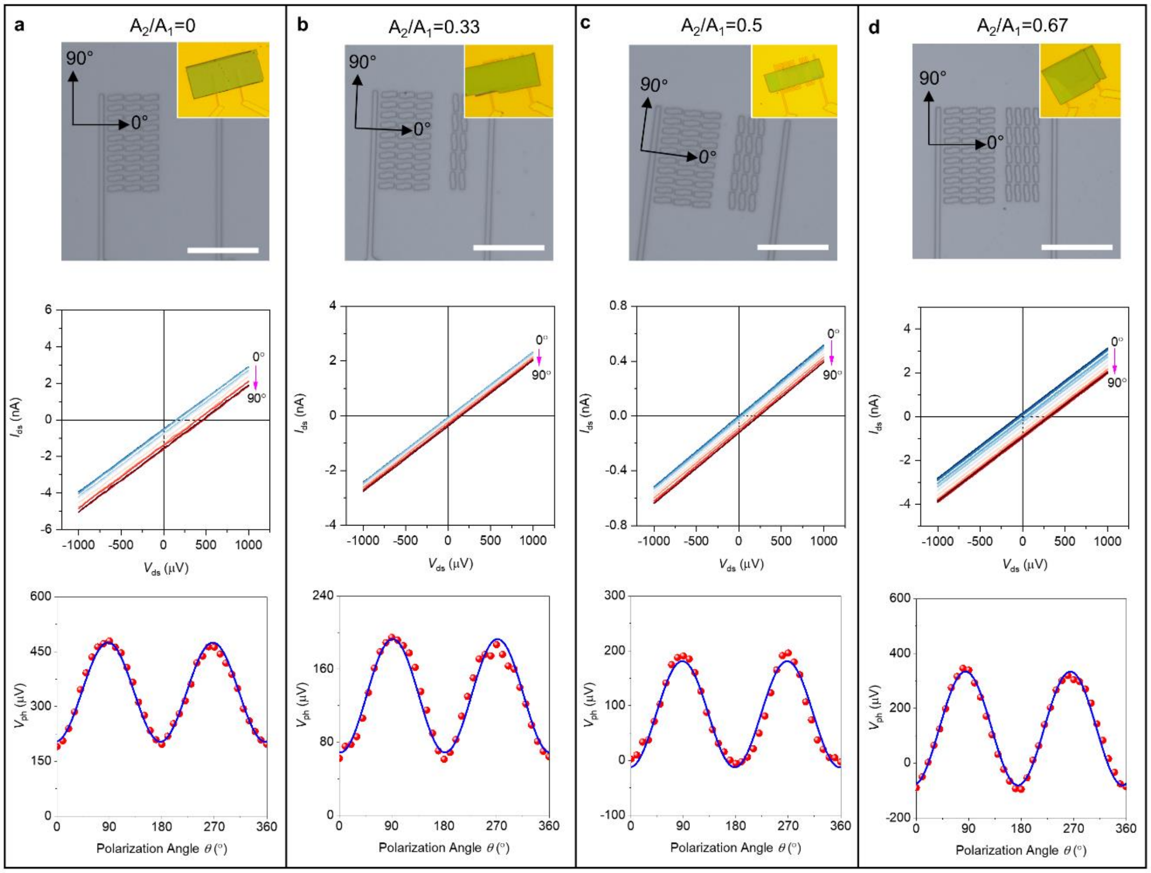

Supplementary Figure 13. Optical images and measured I_{ds}-V_{ds} curves and linear polarization responses of devices with different A_2/A_1 ratios and a fixed orientation angle α of 90°. a, A_2/A_1=0. **b**, A_2/A_1=0.33. **c**, A_2/A_1=0.5. **d**, A_2/A_1=0.67. Scale bars: 20 μm. The light polarization angle along the channel direction is set to be 0°.

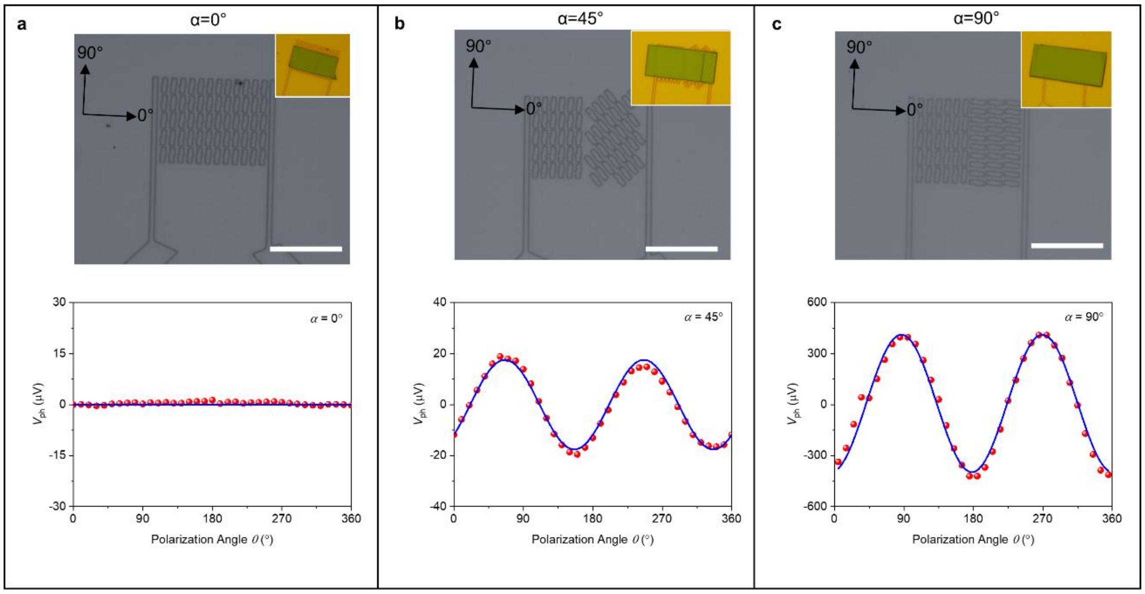

Supplementary Figure 14. Optical images and measured linear polarization responses of devices with different orientation angle α and a fixed A_2/A_1 ratio of 1. a, α=0°. **b,** α=45°. **c,** α=90°. Scale bars: 20 μm. The light polarization angle along the channel direction is set to be 0°.

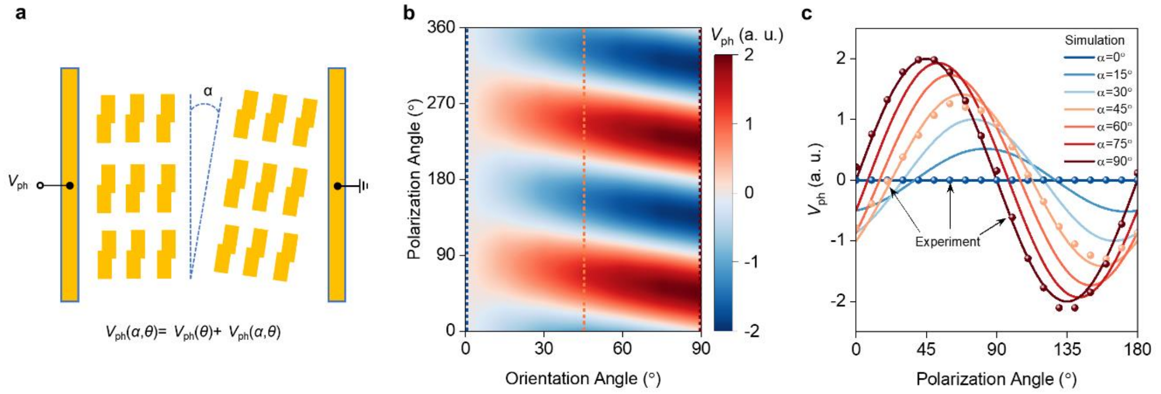

Supplementary Figure 15. Orientation angle dependent linear polarization responses. a, Calculation of the linear polarization angle θ-dependent photoresponses, V_{ph} (α, θ) with different orientation angle (α) of two metamaterials with same distribution area. **b**, Calculation of the linear polarization angle θ-dependent photoresponses V_{ph}(α, θ) with orientation angle. The color bar shows the normalized photoresponses. **c**, Simulated (lines) and measured (symbols) photoresponses of three typical devices with PR=-1 indicated by the dashed lines in **b** with the same colors.

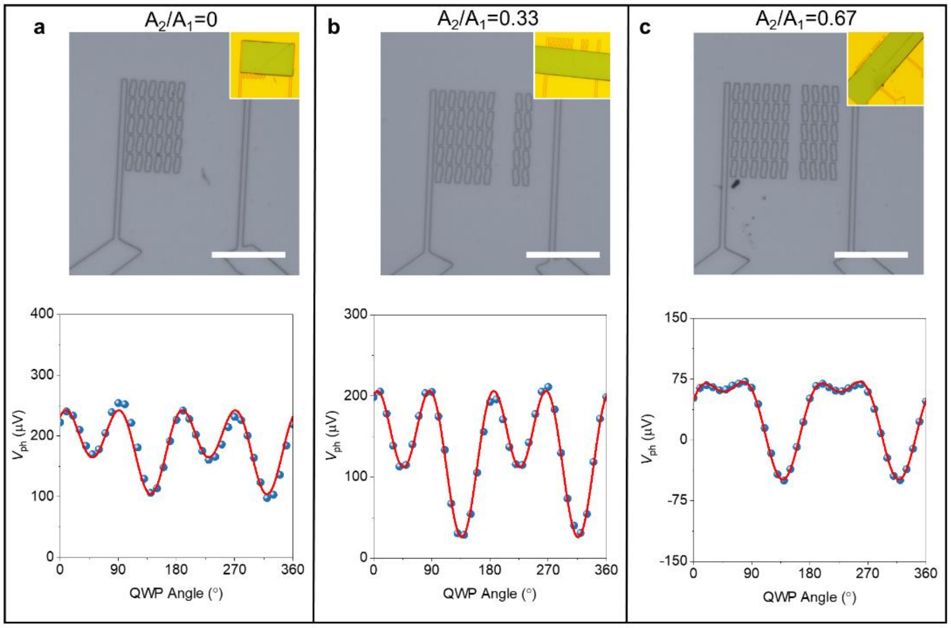

Supplementary Figure 16. Optical images and measured QWP angle dependent responses of devices with different A_2/A_1 ratios. a, A_2/A_1=0. **b,** A_2/A_1=0.33. **c,** A_2/A_1=0.67. Scale bars: 20 µm. The light polarization angle along the channel direction is set to be 0°.

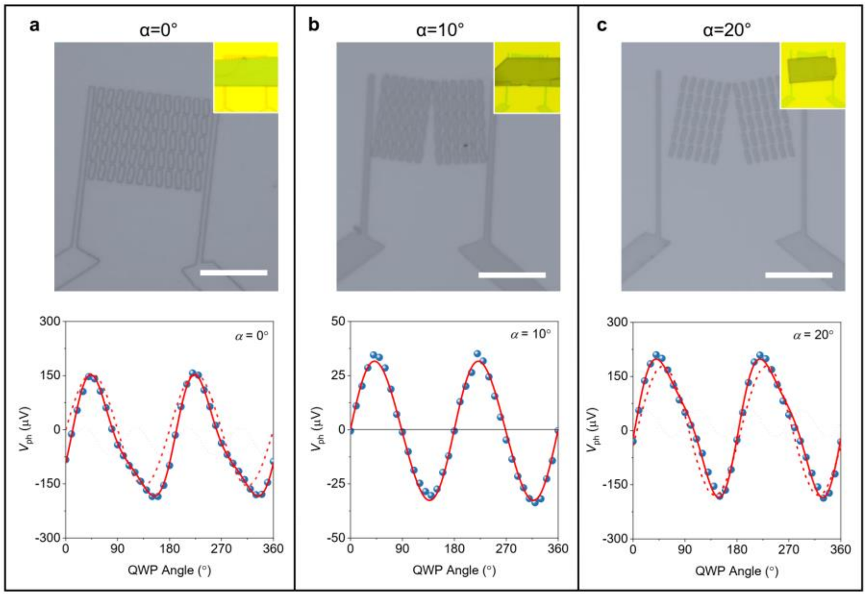

Supplementary Figure 17. Optical images and measured QWP angle dependent responses of devices with different orientation angle α and a fixed A_2/A_1 ratio of 1. a, α=0°. **b,** α=10°. **c,** α=20°. Scale bars: 20 μm. The light polarization angle along the channel direction is set to be 0°.

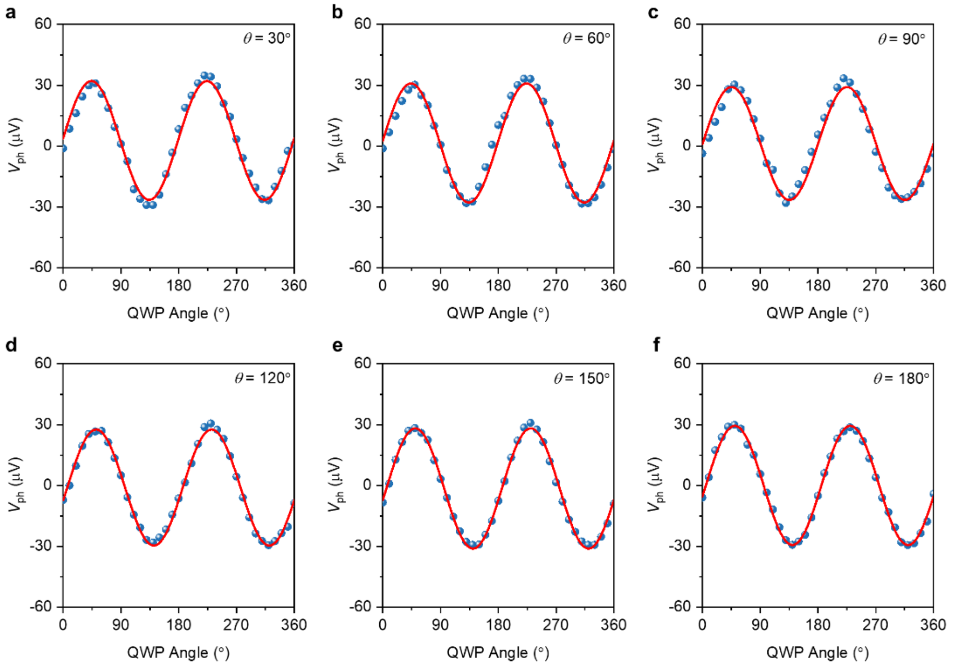

Supplementary Figure 18. QWP angle dependent responses of balanced devices under different linear polarization angle. a-f, The θ changes from 30° to 180° with a step of 30°.

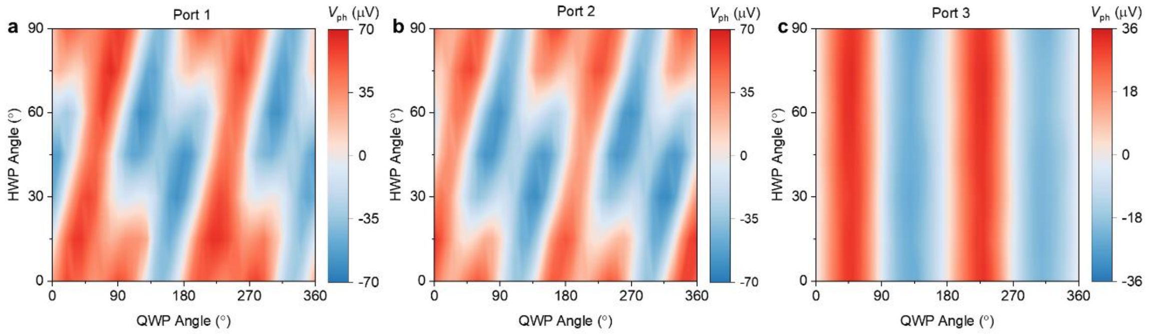

Supplementary Figure 19. Polarization status dependent responses of the three-ports device. a-c, Photoresponse (V_{ph}) of Port 1 (**a**), Port 2 (**b**), and Port 3 (**c**), as a function of HWP angle and QWP angle.

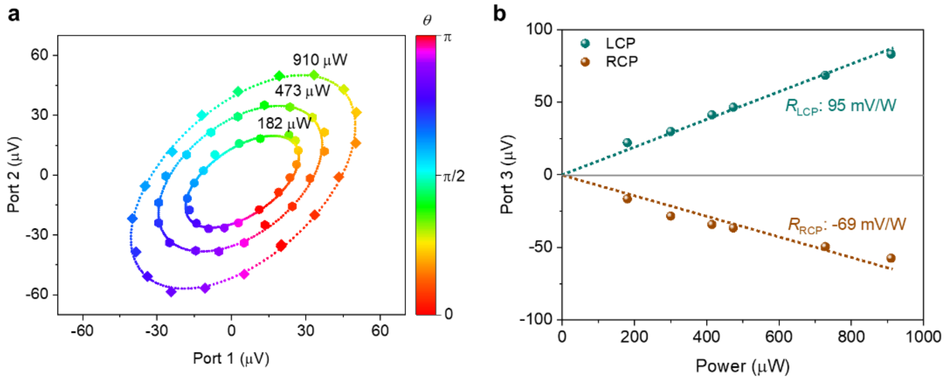

Supplementary Figure 20. Incident light power dependent responses of the three-ports device. a, Photoresponse (V_{ph}) of Port 1 and Port 2 as a function of linear polarization angle (θ) under different incident light powers. **b**, Photoresponse (V_{ph}) as a function of Port 3 for LCP and RCP light under different laser powers at the device.

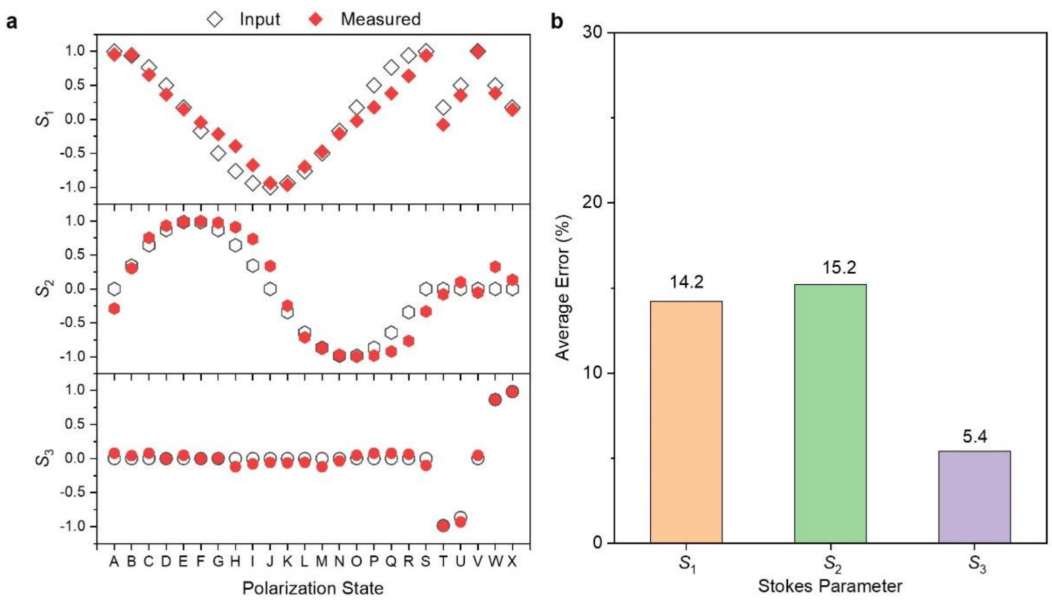

Supplementary Figure 21. The polarimetry accuracy of the three-ports device. a, The retrieved Stokes parameters with different polarization states. The hollow marks indicate input polarization states, and solid marks indicate measured polarization states. **b**, The calculated average errors of three Stokes parameters (S_1, S_2, and S_3).

Supplementary Table 1. Structure parameters of metamaterials with different absorption wavelength peaks.

	Wavelength (nm)	P_1 (nm)	P_2 (nm)	L_1 (nm)	L_2 (nm)	W_1 (nm)	W_2 (nm)	D (nm)
M_1	4500	4200	2500	1950	1400	1150	900	200
M_2	5300	5100	2600	2300	1700	1360	1100	200
M_3	6000	5600	2700	2700	2000	1570	1300	200
M_4	7000	6600	3100	3300	2500	2000	1600	230
M_5	8000	7600	4100	4000	3000	2600	2100	270

Supplementary Table 2. Comparison of linear polarization sensitive photodetectors

<table>
<tr><th colspan="2">Origin of Anisotropy</th><th>Materials</th><th>Wavelength</th><th>Responsivity</th><th>Response time</th><th>PR</th><th>Ref.</th></tr>
<tr><td rowspan="10">Natural Materials</td><td rowspan="7">Intrinsic Crystal Structure</td><td>SbI_3/Sb_2O_3</td><td>450 nm</td><td>1.11 mA/W</td><td>200 ms</td><td>3.14</td><td>[1]</td></tr>
<tr><td>GeSe</td><td>532 nm</td><td>4.25 A/W</td><td>/</td><td>1.09</td><td>[2]</td></tr>
<tr><td>$Gr/PdSe_2/Ge$</td><td>980 nm</td><td>691 mA/W</td><td>92.5 µs</td><td>91.2</td><td>[3]</td></tr>
<tr><td>BP</td><td>1200 nm</td><td>0.35 mA/W</td><td>/</td><td>3.5</td><td>[4]</td></tr>
<tr><td>BP/WSe_2</td><td>1550 nm</td><td>40 mA/W</td><td>0.8 ms</td><td>5.88</td><td>[5]</td></tr>
<tr><td>BP/MoS_2</td><td>3500 nm</td><td>~ 1 A/W</td><td>4 µs</td><td>22</td><td>[6]</td></tr>
<tr><td>$PdSe_2$</td><td>4600 nm</td><td>21.6 V/W</td><td>51.9 µs</td><td>2.06</td><td>[7]</td></tr>
<tr><td rowspan="3">External Geometric Structure</td><td>$CdSe_2$ nanowires</td><td>400 nm</td><td>0.3 A/W</td><td>/</td><td>1.13</td><td>[8]</td></tr>
<tr><td>$CH_3NH_3PbI_3$ nanowires</td><td>500 nm</td><td>45 A/W</td><td>131 ns</td><td>-2.39</td><td>[9]</td></tr>
<tr><td>1D $CsPbBr_3$ arrays</td><td>513 nm</td><td>1000 A/W</td><td>23.4 µs</td><td>2.6</td><td>[10]</td></tr>
<tr><td colspan="2" rowspan="7">Artificial Materials</td><td>Gr/grating antenna</td><td>400-800 nm</td><td>5 V/W</td><td>/</td><td>3</td><td>[11]</td></tr>
<tr><td>BP/bowtie antenna</td><td>1550 nm</td><td>14.2 mA/W</td><td>90 µs</td><td>8.7</td><td>[12]</td></tr>
<tr><td>Gr/bowtie antenna</td><td>6-7 µm</td><td>15 mA/W</td><td>17 ns</td><td>~1.5</td><td>[13]</td></tr>
<tr><td>Gr/nano-stripes</td><td>3-20 µm</td><td>0.6 A/W</td><td>7 ps</td><td>~6</td><td>[14]</td></tr>
<tr><td>Gr/T-shaped antenna</td><td>4 µm</td><td>36.3 mA/W</td><td><100 µs</td><td>-1</td><td>[15]</td></tr>
<tr><td>Gr/tapered antenna</td><td>3.6-4.1 µm</td><td>15.6 V/W</td><td>667 ns</td><td>$1 \rightarrow +\infty$
$-\infty \rightarrow -1$</td><td>[16]</td></tr>
<tr><td>$PdSe_2$/Z-shaped antenna</td><td>5.3 µm</td><td>3.6 V/W</td><td>76 µs</td><td>$1 \rightarrow +\infty$
$-\infty \rightarrow -1$</td><td>This work</td></tr>
</table>

Supplementary Table 3. Comparison of circular polarization sensitive photodetectors

Origin of Chirality		Materials	Wavelength	Responsivity	Response time	*g*-factor	Ref.
Natural Materials	Chiral Absorption	1-aza[6]helicene	365 nm	0.1 µA/W	2.6 ms	1.93	[17]
		(α-PEA)PbI_3	395 nm	797 mA/W	~6.7 ms	0.1	[18]
		(NEA) PbI_3	395 nm	280 mA/W	/	1.85	[19]
		(NEA)2$(MA)_{n-1}Pb_nI_{3n+1}$	405 nm	15.7 A/W	/	0.15	[20]
		$[(R)\text{-bMPA}]_4AgBiI_8$	400-550 nm	22 µA/W	0.96 s	0.23	[21]
		$(\beta\text{-MPA})_2MAPb_2I_7$	500-600 nm	1.1 A/W	2.1 ms	0.11	[22]
		$(\alpha\text{-PEA})_2PbI_4$	532 nm	47.1 A/W	347 µs	0.13	[23]
		ProSQ-C_6:PCBM	550 nm	64.5 mA/W	/	0.1	[24]
		bis[60]PCBM	300-700 nm	/	43 ms	1.27	[25]
		PODTPPD-BT	750-1000 nm	300 A/W	0.31 s	1.9	[26]
	Spin PGE	Bi_2Te_2Se	633 nm	1.66 mA/W	/	1.55	[27]
		$Bi_{1.5}Sb_{0.5}Te_{1.8}Se_{1.2}$	532 nm	/	/	1.74	[28]
	Inverse Spin Hall Effect	Au/InP	532 nm	/	/	∞	[29]
		Pt/GaAs	670 nm	/	/	∞	[30]
	Spin-Dependent Recombination	$GaAs_{1-x}N_x$	640-840 nm	/	/	0.075	[31]
		$GaAs_{1-x}N_x$	852 nm	/	/	0.05	[32]
Artificial Materials		X-shaped Al	500-600 nm	92.5 mA/W	/	0.8	[33]
		Chiral Ag Cavity	660 nm	/	6.2 ms	1.6	[34]
		Ag/Au/$MoSe_2$	500-900 nm	2.46 mA/W	< 100 ms	0.38	[35]
		Z-shaped Ag	1.2-1.7 µm	2.2 mA/W	/	1.1	[36]
		Chiral Au/Si	14.-1.7 µm	27 mA/W	/	1.27	[37]
		Chiral Au/Gr	3.6-4.0 µm	0.8 µA/W	/	1.5	[38]
		Chiral Au/$PdSe_2$	5.3 µm	95 mV/W	76 µs	0.44→∞	This work

Supplementary References

1. Xiao M, et al. Symmetry-Reduction Enhanced Polarization-Sensitive Photodetection in Core–Shell SbI_3/Sb_2O_3 van der Waals Heterostructure. *Small* **16**, 1907172 (2020).

2. Wang X, et al. Short-Wave Near-Infrared Linear Dichroism of Two-Dimensional Germanium Selenide. *J. Am. Chem. Soc.* **139**, 14976-14982 (2017).

3. Wu D, et al. Highly Polarization-Sensitive, Broadband, Self-Powered Photodetector Based on Graphene/$PdSe_2$/Germanium Heterojunction. *ACS Nano* **13**, 9907-9917 (2019).

4. Yuan H, et al. Polarization-sensitive broadband photodetector using a black phosphorus vertical p–n junction. *Nat. Nanotech.* **10**, 707-713 (2015).

5. Ye L, et al. Highly polarization sensitive infrared photodetector based on black phosphorus-on-WSe_2 photogate vertical heterostructure. *Nano Energy* **37**, 53-60 (2017).

6. Bullock J, et al. Polarization-resolved black phosphorus/molybdenum disulfide mid-wave infrared photodiodes with high detectivity at room temperature. *Nat. Photonics* **12**, 601-607 (2018).

7. Dai M, et al. High-Performance, Polarization-Sensitive, Long-Wave Infrared Photodetection via Photothermoelectric Effect with Asymmetric van der Waals Contacts. *ACS Nano* **16**, 295-305 (2022).

8. Singh A, et al. Polarization-Sensitive Nanowire Photodetectors Based on Solution-Synthesized CdSe Quantum-Wire Solids. *Nano Lett.* **7**, 2999-3006 (2007).

9. Cao G, Zhang H, Chen G, Li X. Ambipolar Self-Driving Polarized Photodetection. *ACS Photonics* **8**, 2459-2465 (2021).

10. Feng J, et al. Crystallographically Aligned Perovskite Structures for High-Performance Polarization-Sensitive Photodetectors. *Adv. Mater.* **29**, 1605993 (2017).

11. Echtermeyer TJ, et al. Strong plasmonic enhancement of photovoltage in graphene. *Nat. Commun.* **2**, 458 (2011).

12. Venuthurumilli PK, Ye PD, Xu X. Plasmonic Resonance Enhanced Polarization-Sensitive Photodetection by Black Phosphorus in Near Infrared. *ACS Nano* **12**, 4861-4867 (2018).

13. Castilla S, et al. Plasmonic antenna coupling to hyperbolic phonon-polaritons for sensitive and fast mid-infrared photodetection with graphene. *Nat. Commun.* **11**, 4872 (2020).

14. Cakmakyapan S, Lu PK, Navabi A, Jarrahi M. Gold-patched graphene nano-stripes for high-responsivity and ultrafast photodetection from the visible to infrared regime. *Light: Sci. Appl.* **7**, 20 (2018).

15. Wei J, et al. Zero-bias mid-infrared graphene photodetectors with bulk photoresponse and calibration-free polarization detection. *Nat. Commun.* **11**, 6404 (2020).

16. Wei J, Xu C, Dong B, Qiu C-W, Lee C. Mid-infrared semimetal polarization detectors with configurable polarity transition. *Nat. Photonics* **15**, 614-621 (2021).

17. Yang Y, da Costa RC, Fuchter MJ, Campbell AJ. Circularly polarized light detection by a chiral organic semiconductor transistor. *Nat. Photonics* **7**, 634-638 (2013).

18. Chen C, et al. Circularly polarized light detection using chiral hybrid perovskite. *Nat. Commun.* **10**, 1927 (2019).

19. Ishii A, Miyasaka T. Direct detection of circular polarized light in helical 1D perovskite-based photodiode. *Sci. Adv.* **6**, eabd3274 (2020).

20. Liu T, et al. High Responsivity Circular Polarized Light Detectors based on Quasi Two-Dimensional Chiral Perovskite Films. *ACS Nano* **16**, 2682–2689 (2022).

21. Li D, et al. Chiral Lead-Free Hybrid Perovskites for Self-Powered Circularly Polarized Light Detection. *Angew. Chem., Int. Ed.* **60**, 8415-8418 (2021).

22. Wang L, et al. A Chiral Reduced-Dimension Perovskite for an Efficient Flexible Circularly Polarized Light Photodetector. *Angew. Chem., Int. Ed.* **59**, 6442-6450 (2020).

23. Zhao Y, et al. Chiral 2D-Perovskite Nanowires for Stokes Photodetectors. *J. Am. Chem. Soc.* **143**, 8437-8445 (2021).

24. Schulz M, et al. Chiral Excitonic Organic Photodiodes for Direct Detection of Circular Polarized Light. *Adv. Funct. Mater.* **29**, 1900684 (2019).

25. Shi W, et al. Fullerene Desymmetrization as a Means to Achieve Single-Enantiomer Electron Acceptors with Maximized Chiroptical Responsiveness. *Adv. Mater.* **33**, 2004115 (2021).

26. Han H, et al. High-Performance Circularly Polarized Light-Sensing Near-Infrared Organic Phototransistors for Optoelectronic Cryptographic Primitives. *Adv. Funct. Mater.* **30**, 2006236 (2020).

27. Huang S, Xu X. Optical Chirality Detection Using a Topological Insulator Transistor. *Adv. Opt. Mater.* **9**, 2002210 (2021).

28. Sun X, et al. Topological insulator metamaterial with giant circular photogalvanic effect. *Sci. Adv.* **7**, eabe5748 (2021).

29. Khamari SK, Porwal S, Oak SM, Sharma TK. A spin-optoelectronic detector for the simultaneous measurement of the degree of circular polarization and intensity of a laser beam. *Appl. Phys. Lett.* **107**, 072108 (2015).

30. Ando K, et al. Photoinduced inverse spin-Hall effect: Conversion of light-polarization information into electric voltage. *Appl. Phys. Lett.* **96**, 082502 (2010).

31. Ibarra-Sierra VG, et al. Polarization-Sensitive Photodetector Based on $GaAs_{1-x}N_x$. *Phys. Rev. Appl.* **15**, 064040 (2021).

32. Joshya RS, et al. Chiral Photodetector Based on GaAsN. *Adv. Funct. Mater.* **31**, 2102003 (2021).

33. Shi X, et al. Circularly Polarized Light Photodetector Based on X-Shaped Chiral Metamaterial. *IEEE Sens. J.* **18**, 9203-9206 (2018).

34. Kim H, Ryoul Park K, Kim C. High-performance circular-polarization-sensitive organic photodetectors based on a chiral plasmonic nanocavity. *Opt. Express* **28**, 1805-1816 (2020).

35. Jiang Q, et al. Ultrathin circular polarimeter based on chiral plasmonic metasurface and monolayer $MoSe_2$. *Nanoscale* **12**, 5906-5913 (2020).

36. Li W, et al. Circularly polarized light detection with hot electrons in chiral plasmonic metamaterials. *Nat. Commun.* **6**, 8379 (2015).

37. Xiao W, Shi X, Zhang Y, Peng W, Zeng Y. Circularly polarized light detector based on 2D embedded chiral nanostructures. *Phys. Scr.* **94**, 085501 (2019).

38. Peng J, Cumming BP, Gu M. Direct detection of photon spin angular momentum by a chiral graphene mid-infrared photodetector. *Opt. Lett.* **44**, 2998-3001 (2019).
